# A subset of octopaminergic neurons that promotes feeding initiation in *Drosophila melanogaster*

**DOI:** 10.1371/journal.pone.0198362

**Published:** 2018-06-27

**Authors:** Hyesoo Youn, Colleen Kirkhart, Justine Chia, Kristin Scott

**Affiliations:** 1 Department of Molecular and Cell Biology, University of California, Berkeley, Berkeley, CA, United States of America; 2 Helen Wills Neuroscience Institute, University of California, Berkeley, Berkeley, CA, United States of America; AgroParisTech, FRANCE

## Abstract

Octopamine regulates feeding behavioral responses in *Drosophila melanogaster*, however the molecular and circuit mechanisms have not been fully elucidated. Here, we investigated the role of a subset of octopaminergic neurons, the OA-VPM4 cluster, in sucrose acceptance behavior. Thermogenetic activation of Gal4 lines containing OA-VPM4 promoted proboscis extension to sucrose, while optogenetic inactivation reduced extension. Anatomically, the presynaptic terminals of OA-VPM4 are in close proximity to the axons of sugar-responsive gustatory sensory neurons. Moreover, RNAi knockdown of a specific class of octopamine receptor, OAMB, selectively in sugar-sensing gustatory neurons decreased the behavioral response to sucrose. By calcium imaging experiments, we found that application of octopamine potentiates sensory responses to sucrose in satiated flies. Taken together, these findings suggest a model by which OA-VPM4 promotes feeding behavior by modulating the activity of sensory neurons.

## Introduction

Animals must constantly adjust their feeding behaviors to fulfill their nutritional needs. Regulatory mechanisms for food intake are crucial to balance caloric consumption, energy expenditure and the body weight of an organism. Despite their importance, the neural circuits and molecular mechanisms underpinning how internal physiological state modulates feeding behavior have yet to be fully elucidated.

The fruit fly *Drosophila melanogaster* is an excellent model system to examine modulatory mechanisms that regulate food intake. The simplicity of the fly nervous system (100,000 neurons versus 10–100 billion in mammals) as well as the powerful molecular, genetic and functional approaches available in this organism provide the opportunity to examine feeding regulation with cellular resolution. Studying neural and molecular mechanisms in animals with compact nervous systems may provide valuable insights into shared features of feeding regulation.

In *Drosophila*, feeding initiation begins with the proboscis extension response (PER). When gustatory receptor neurons (GRNs) on the legs detect sugar, the fly extends its proboscis to initiate feeding [1]. The probability of proboscis extension to sugar stimuli increases with both starvation time and sugar concentration, and decreases when bitter compounds are present. Thus, the proboscis extension response is regulated by hunger and satiety as well as by external gustatory stimuli.

The neural circuits that link taste detection to proboscis extension are beginning to be elucidated. GRNs are found in chemosensory sensilla on the proboscis, internal mouthparts and legs [2]. Each sensillum contains two or four GRNs that recognize different taste modalities, including bitter, sugar, water and pheromones [3,4]. GRNs from the proboscis, mouthparts and legs project to the subesophageal zone (SEZ) of the fly brain [3,4]. Motor neurons and interneurons that drive proboscis extension and feeding also reside in the SEZ [5–11], suggesting that there may be local SEZ circuits processing and directing behavior from taste detection to proboscis extension.

These feeding circuits must be highly regulated to adjust feeding decisions based on nutritional needs. One site of regulation is the sensory neuron. Elegant studies have revealed that neuromodulatory cascades impinge on GRNs to modulate activity in nutrient-deprived conditions [12–15]. For example, dopamine potentiates the response of sugar-sensitive GRNs to promote consumption when the animal is food deprived [12]. Similarly, octopamine (OA) alters the sensitivity of bitter-sensing GRNs, increasing their response in a fed state, to enhance rejection of bitter compounds [14]. Altering the gain of sensory responses serves to promote sensitivity to sugars and decrease sensitivity to bitter compounds upon food deprivation, actions that increase feeding probability.

In addition to its role in modulating bitter GRN responses, OA influences responses to other chemosensory cues. For example, OA neurons form direct contacts with pheromone-sensing chemosensory neurons and influence aggression [16]. OA also regulates the response to sugars and promotes feeding behaviors in starved states. Larvae lacking a critical enzyme for OA biosynthesis, tyramine *β-*hydroxylase (T*β*h), do not show increased feeding upon starvation [17] and adults show decreased proboscis extension to sugar in the starved state [18]. In addition, OA promotes starvation-induced increases in foraging [19]. These studies argue that OA promotes responses to sweet compounds in deprived states. However, the mechanisms by which OA modulates sweet responses and sugar feeding are unknown.

Here, to examine how OA modulates the response to sucrose, we characterize a subset of octopaminergic neurons, the OA-VPM4 cluster [20], and find that these neurons promote proboscis extension to sugar. RNAi mediated knockdown of the enzymes required for OA synthesis specifically in OA-VPM4 decreased proboscis extension to sucrose, suggesting feeding regulation through these neurons requires OA. OA-VPM4 is anatomically well positioned to regulate activity of the sugar-sensing GRNs, as their axonal arbors intermingle. Moreover, RNAi knockdown of the OA receptor, OAMB, in sugar sensory neurons decreased proboscis extension to sucrose. These studies demonstrate that OA-VPM4 promotes feeding initiation and suggest that it may modulate activity of sugar-sensing GRNs, providing mechanistic insight into the process of feeding and its regulation.

## Results

*Drosophila* octopaminergic neurons are a diverse class, containing at least 27 cell types [20]. Recent studies have shown that the OA-VL cluster of OA neurons modulates bitter GRN function, increasing their activity in the fed state [14]. Because OA is also required for responses to sugars [18], we hypothesized that additional classes of OA neurons might contribute to starvation-induced feeding increases. We examined the role of a cluster of ventral paired median OA neurons, OA-VPM4, because they have rich projections in the subesophageal zone (SEZ), a region that participates in taste information processing [20,21].

**Fig 1:**
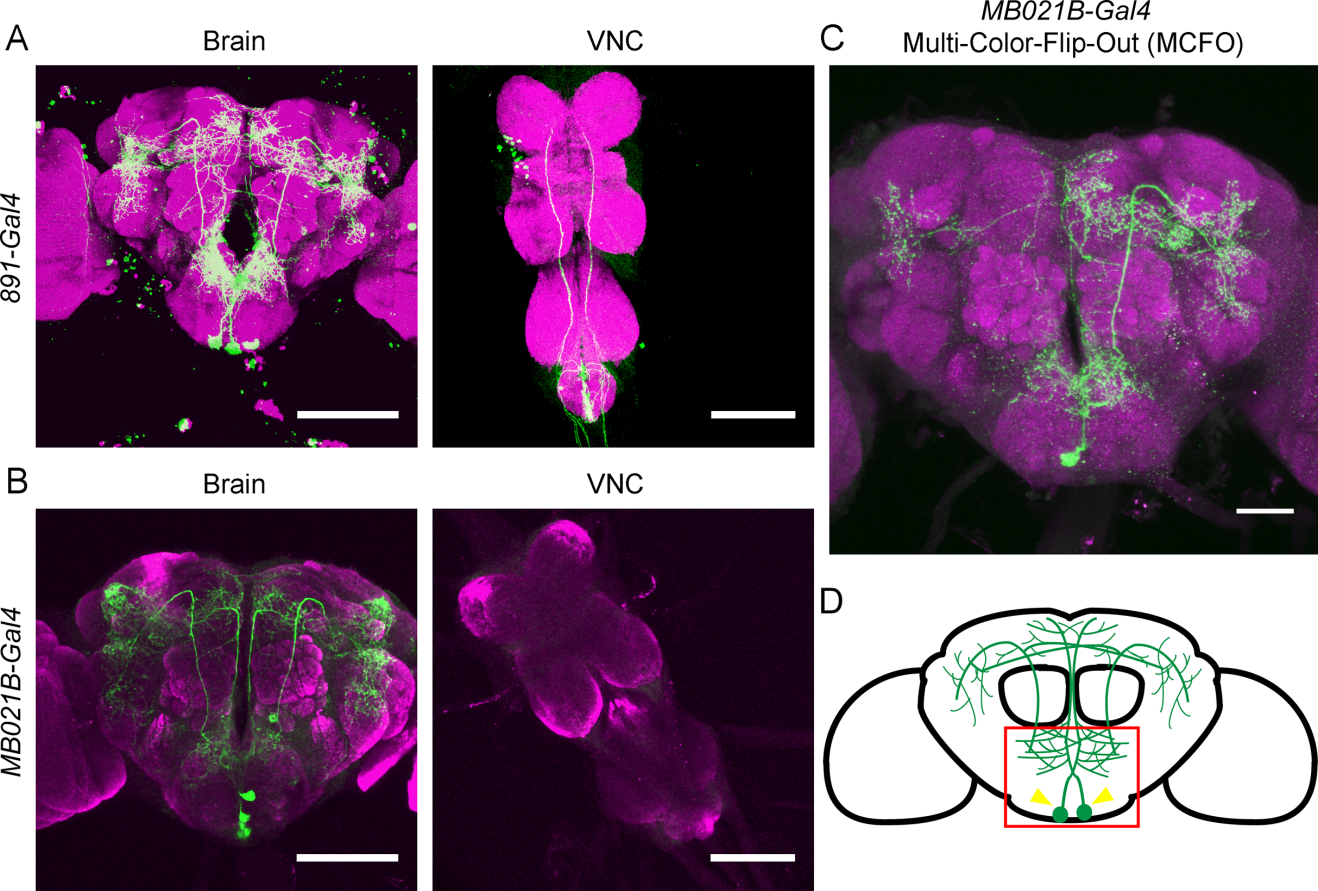
Anatomy of OA-VPM4. (A, B) Expression pattern of *891-Gal4* (A), and *MB021B-Gal4* (B), containing the OA-VPM4 cluster (*UAS-mCD8GFP*, green). Scale = 100 μm. (C) Single labeled OA-VPM4 cell generated by Multi-Color-Flip-Out (MCFO) using *MB021B-Gal4*. Scale = 50μm. (D) Schematic showing OA-VPM4 arbors, with arrows denoting cell bodies and box showing SEZ. Magenta indicates neuropil, labeled with nc82 (A-D).

Two Gal4 lines, *891-Gal4* and *MB021B-Gal4*, have been previously reported to drive expression in OA-VPM4 and a few additional neurons [21,22]. *891-Gal4* drives expression in OA-VPM4 as well as a few protocerebral neurons and VNC projections (Fig 1A*)*. *MB021B-Gal4* drives expression in OA-VPM4 and two additional ventral SEZ neurons (Fig 1B*)*. The cell bodies of OA-VPM4 are located lateral to the midline and their processes separate from median tracts toward the ventral esophagus. The arbors project not only within the SEZ, but also extend broadly throughout the protocerebrum, including the gamma lobe of the mushroom body. Single-cell clones of OA-VPM4, using the multi-color-flip-out (MCFO) approach as a stochastic labeling strategy, showed that OA-VPM4 indeed has ipsilateral and contralateral projections in the SEZ and protocerebrum, with more ipsilateral than contralateral fibers (Fig 1C). The only neurons shared between *891-Gal4* line and *MB021B-Gal4* line are OA-VPM4 based on visual inspection. The arborization pattern of OA-VPM4 suggested the possibility that OA-VPM4 might participate in relaying taste detection from the SEZ to the higher brain (Fig 1D).

**Fig 2:**
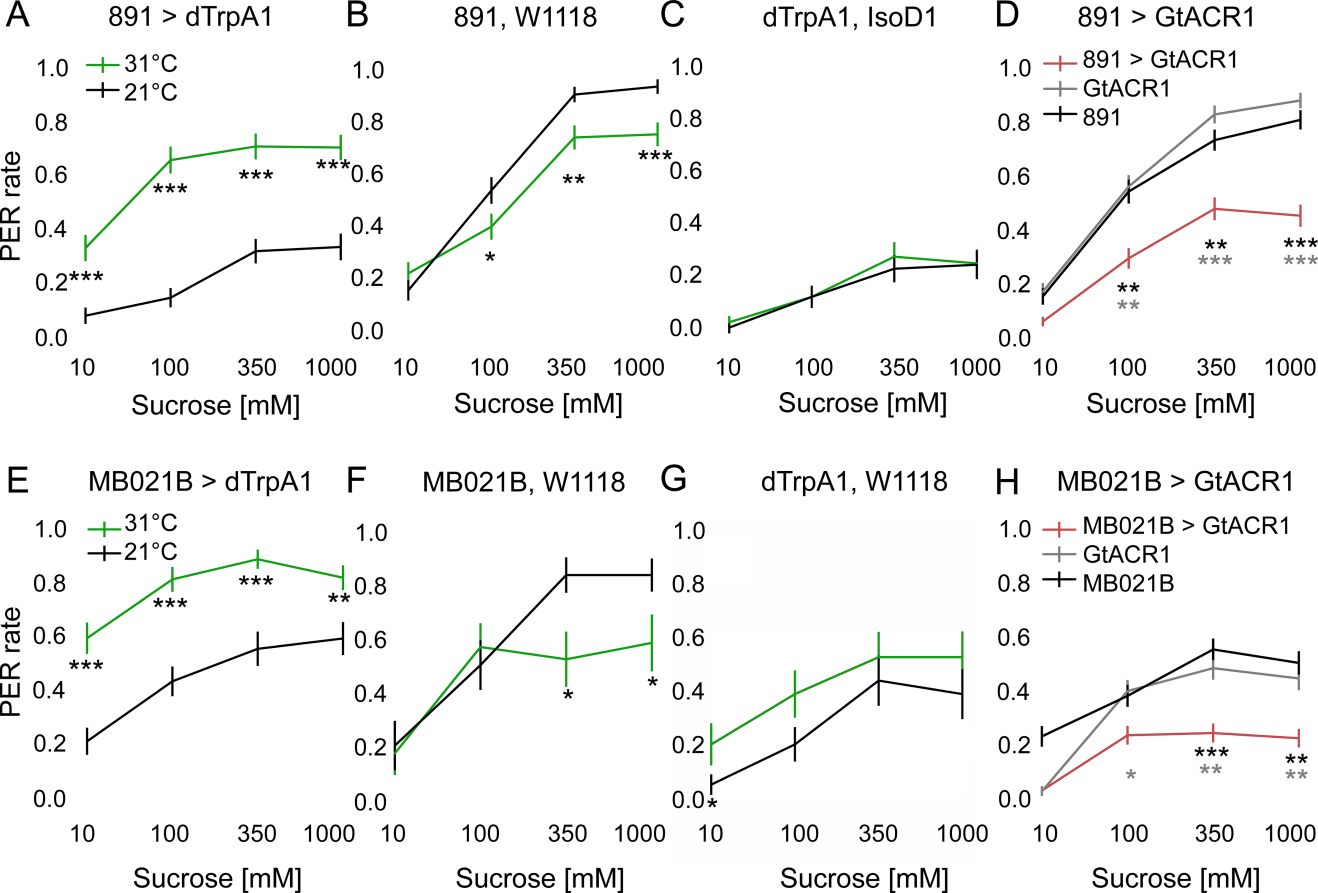
Inducible activation or inactivation of OA-VPM4 alters the threshold for proboscis extension. (A-C, E-G) PER was tested to four different concentration of sucrose (10mM, 100mM, 350mM, 1M) while neurons in *891-Gal4* (A) or *MB021B-Gal4* (E) were thermogenetically activated with *UAS-dTrpA1*. Flies starved for 24 hours were heated to 31°C for ~5 min before and during testing to activate dTrpA1, or were kept at room temperature (21°C) as controls. Genetic controls for each Gal4 line and *UAS-dTrpA1* line were also tested in the same condition (B, C, F, G). n = 86 (A), 86 (B), 67 (C), 60 (E), 22 (F) and 26 (G) flies, mean ± SEM, Mann-Whitney-U test, *p<0.05, **p<0.01, ***p<0.001. (D, H) PER was tested to four different concentration of sucrose (10mM, 100mM, 350mM, 1M) while neurons in *891-Gal4* (D) or *MB021B-Gal4* (H) were optogenetically silenced with the chloride channel, GtACR1. Flies starved for 24 hours were stimulated with 530nm light to activate GtACR1. n = 118 (D) and 122 (H) flies, mean ± SEM, Kruskal-Wallis 1-way ANOVA with Bonferroni correction. *p<0.05, **p<0.01, ***p<0.001. Pairwise comparisons to each control, colors of asterisks indicate which genetic controls were compared to the experimental group.

The proboscis extension response (PER) is an innate gustatory-driven behavior. When GRNs on the legs detect sugar, the fly extends its proboscis in order to initiate feeding, whereas inclusion of bitter compounds suppresses proboscis extension [1]. Previous studies showed that *Tβh* mutants, which lack an enzyme required for OA biosynthesis, show decreased proboscis extension to sugars, suggesting that OA is required for PER modulation [18]. We therefore examined if genetically activating the OA-VPM4 cluster is sufficient to modulate PER. To do so, we acutely activated OA-VPM4 neurons using dTrpA1, a temperature-sensitive cation channel [23]. Flies expressing *UAS-dTrpA1* under the control of the two OA-VPM4 containing Gal4 lines were either heated to 31°C to activate dTrpA1 or kept at room temperature 21°C as controls. Flies did not spontaneously show PER upon dTrpA1 activation. However, when sucrose was presented to the tarsi (10mM, 100mM, 350mM, 1M, presented serially in order), flies showed enhanced PER upon dTrpA1 activation to all sucrose concentrations (Fig 2A–2C and 2E–2G). Some genetic controls showed decreased responses at 31°C (Fig 2B and 2F), but these heat-dependent decreases contrast with the heat-dependent increases seen in experimental groups. These results argue that increased activity of OA-VPM4 is sufficient to enhance PER.

Next, we sought to examine whether OA-VPM4 bidirectionally modulates sugar taste sensitivity by testing whether OA-VPM4 neurons are necessary for proboscis extension to sucrose (Fig 2D and 2H). We optogenetically inhibited OA-VPM4 using GtACR1, a light-gated anion channel [24], and examined the effects on PER to different sucrose concentrations applied to tarsi. Interestingly, PER was suppressed significantly when OA-VPM4 neurons were acutely inactivated, compared to genetic controls. Taken together, these results show that activity of OA-VPM4 is both necessary and sufficient for modulating sugar taste sensitivity.

**Fig 3:**
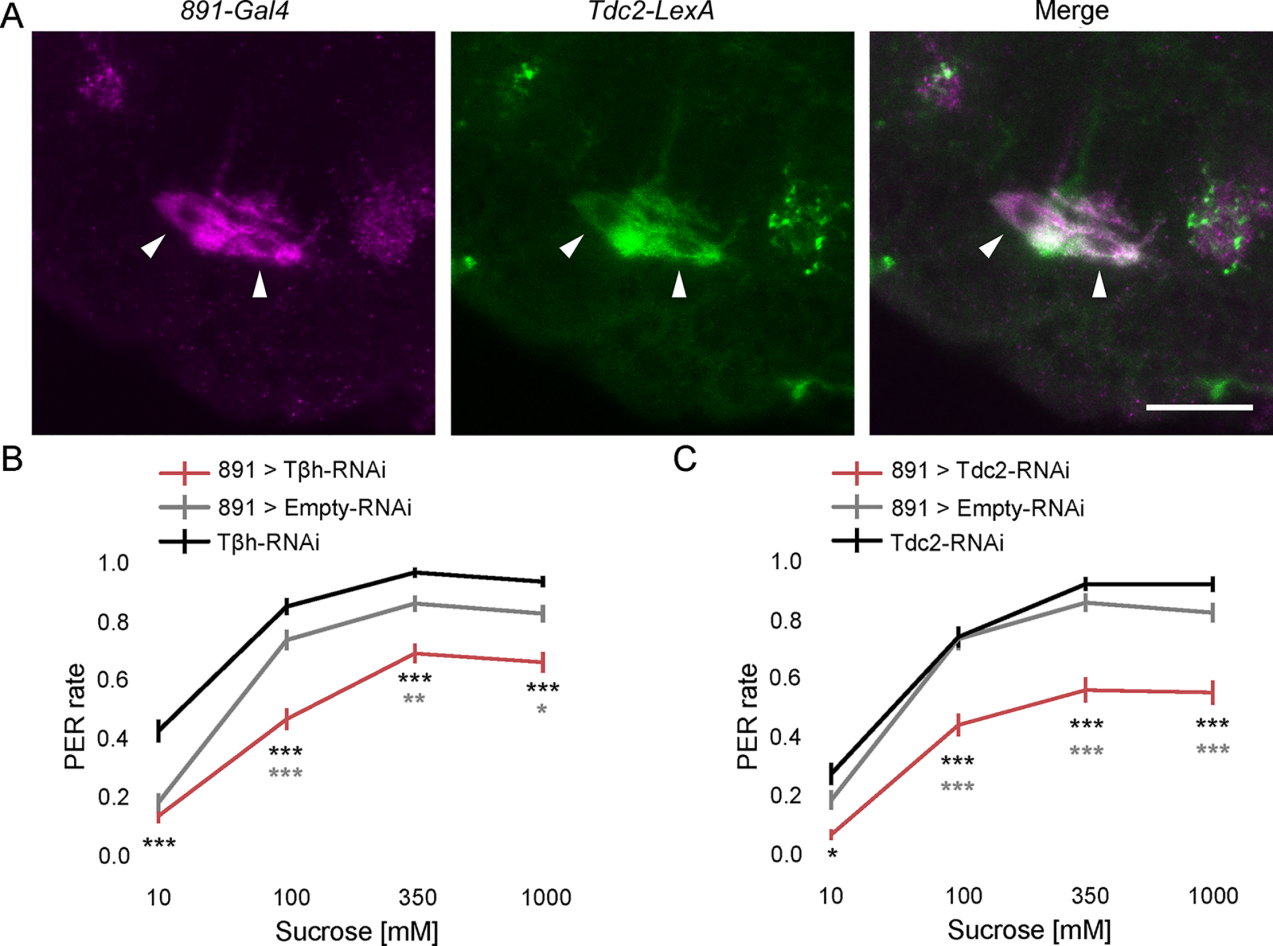
Octopamine is required for proboscis extension modulation by OA-VPM4. (A) Double labeling of neurons in *891-Gal4* (magenta, *CD8*::*tdTomato*) with OA neurons driven by *Tdc2-LexA* (green, *CD2*::*GFP*). Arrows point to OA-VPM4 cell bodies in the SEZ, showing co-labeling with the *Tdc2-LexA* reporter. Scale = 20 μm. (B, C) Flies expressing Tβh RNAi (B) or Tdc2 RNAi (C) in *891-Gal4* neurons, were tested for PER to 10mM, 100mM, 350mM, 1M sucrose. n = 155 (B) and 120 (C) flies, mean ± SEM, Kruskal-Wallis 1-way ANOVA with Bonferroni correction. *p<0.05, **p<0.01, ***p<0.001. Pairwise comparisons to each control, asterisk colors indicate which genetic controls were compared to the experimental group.

We used the *891-Gal4* line to further characterize the role of OA-VPM4 in taste modulation, because it showed the strongest OA-VPM4 labeling, with minimal expression in other neurons. We first examined whether OA-VPM4 neurons indeed express tyrosine decarboxylase 2 (Tdc2), an enzyme required for octopamine synthesis [20,21] by examining co-labeling between *Tdc2-LexA* and *891-Gal4* (Fig 3A and Fig A in S1 Fig). This revealed that OA-VPM4 neurons are co-labeled with *Tdc2-LexA*, arguing that they are indeed octopaminergic. No other OA neurons were detected in the *891-Gal4* line.

To determine if the OA-VPM4 modulation of sugar taste sensitivity requires OA, we knocked down Tdc2 or Tβh, required for OA synthesis [25,26], in *891-Gal4* using RNAi. As OA-VPM4 neurons are the only OA neurons in this Gal4 line, expressing RNAi under *891-Gal4* driver should exclusively decrease OA in OA-VPM4. If OA-VPM4 requires OA for feeding regulation, knocking down these enzymes is predicted to alter sugar taste sensitivity in the flies. Indeed, knocking down either enzyme robustly reduced PER to sucrose (Fig 3B and 3C), arguing that OA-VPM4 requires OA to regulate feeding initiation.

To further determine the role of OA-VPM4 in feeding regulation, we sought to examine how OA-VPM4 neurons enhance sugar taste sensitivity. OA-VPM4 might be a primary neural component of a sensorimotor circuit for proboscis extension, or may modulate activity of PER circuits in response to other cues. To distinguish between these models, we tested whether OA-VPM4 responded to sensory detection of taste compounds. We monitored taste-induced activity in OA-VPM4 by GCaMP6s calcium imaging experiments in live flies stimulated with sucrose delivered to the proboscis (S2 Fig). We did not observe calcium increases upon tastant stimulation, suggesting that sugar-sensing proboscis GRNs do not activate OA-VPM4 and OA-VPM4 is not a primary component of the taste detection circuitry.

**Fig 4:**
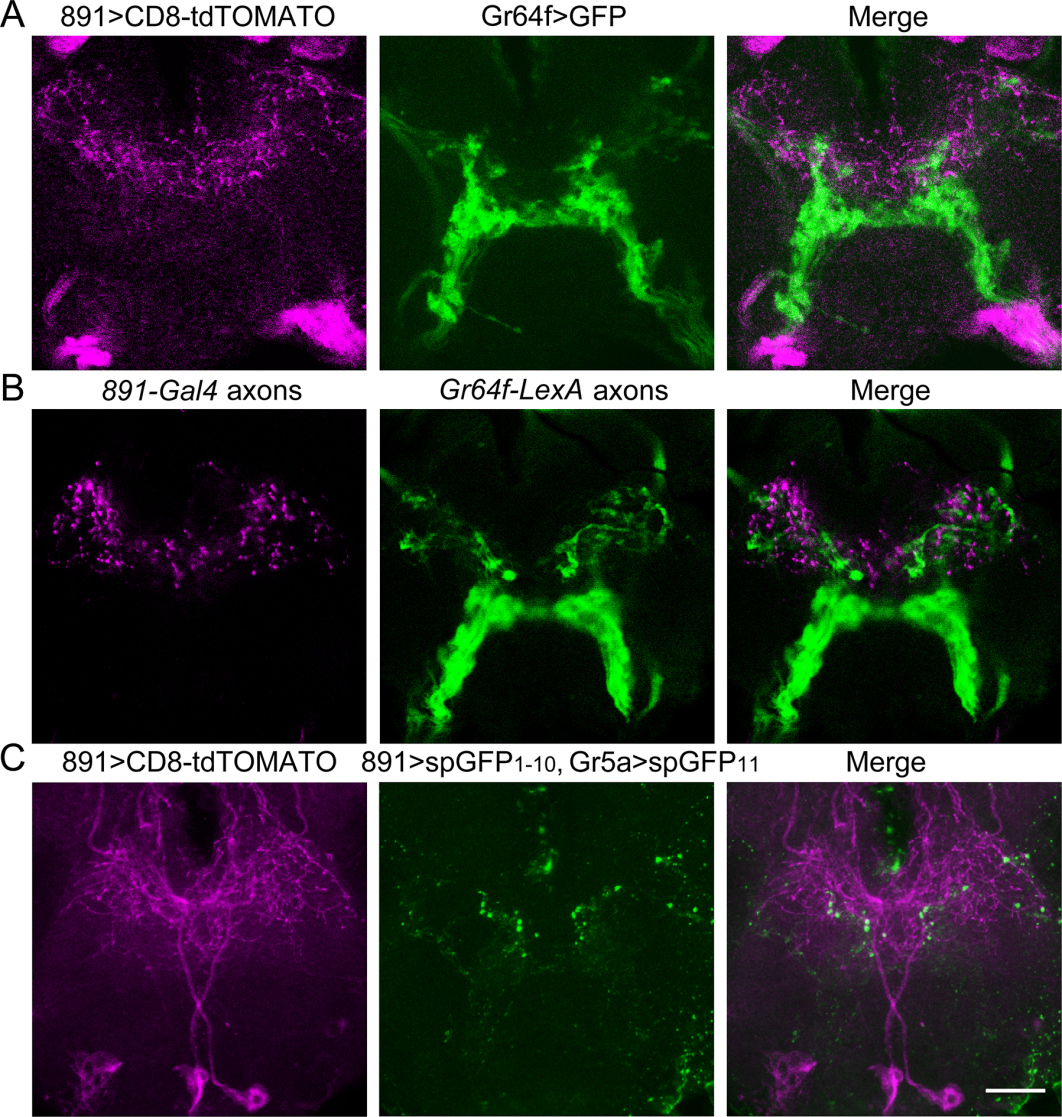
OA-VPM4 axons arborize near sugar sensory fibers. (A) Double labeling of *891-Gal4* (magenta, CD8-tdTOMATO) and sugar-sensing GRNs (green, CD4-GFP) indicates that axons of sugar-sensing GRNs anatomically overlap with neural processes in *891-Gal4*. Shown is SEZ as in Fig 1D, where gustatory axons project. Single optical slice (2 μm) in region of maximum overlap is shown. (B) *891-Gal4* axons (magenta, synaptotagmin::eGFP) and sugar-sensing GRNs (green, CD8-tdTOMATO) overlap in the SEZ. Single optical slice (2 μm) in region of maximum overlap is shown. (C) Reconstitution of GFP in flies expressing spGFP(1–10) in *891-Gal4* (co-labeled with CD8-tdTOMATO, magenta) and spGFP(11) in sugar-sensing GRNs (*Gr5a-LexA*). Scale = 20μm.

An alternate hypothesis is that OA-VPM4 is a regulatory component that modulates responses in PER circuits, thus regulating feeding. Anatomically, OA-VPM4 has rich projections in the SEZ, the main taste-processing center in fly brain. Therefore, we wondered whether OA-VPM4 communicates with gustatory or feeding related SEZ neurons. We tested whether OA-VPM4 arborizes in proximity to sugar-sensing GRNs by double labeling experiments using *891-Gal4* and *Gr64f-LexA or Gr5a-LexA*, both of which mark sugar-sensing GRNs. Interestingly, some OA-VPM4 neurites are in close proximity to sugar-sensing GRN axons (Fig 4A), suggesting possible communication between OA-VPM4 fibers in the SEZ and gustatory projections. Examination of pre- or postsynaptic terminals, using synaptotagmin::eGFP and Denmark::RFP respectively [27,28], revealed that OA-VPM4 has both postsynaptic terminals and presynaptic terminals in the SEZ (Fig 4B and S3 Fig). Moreover, the presynaptic terminals of OA-VPM4, but not the postsynaptic terminals, anatomically overlap with the axons of sugar-sensing GRNs (Fig 4B and S3 Fig). In addition, experiments expressing membrane-tethered complementary split-GFP fragments in OA-VPM4 and sugar-sensing GRNS, (using GFP Reconstitution Across Synaptic Partners; GRASP, [5]), revealed punctate GFP reconstitution, arguing for proximity (Fig 4C). The anatomical studies suggest that OA-VPM4 might feed back onto GRN axons, perhaps by localized octopamine release onto octopamine receptors in gustatory axons, to modulate their responses.

**Fig 5:**
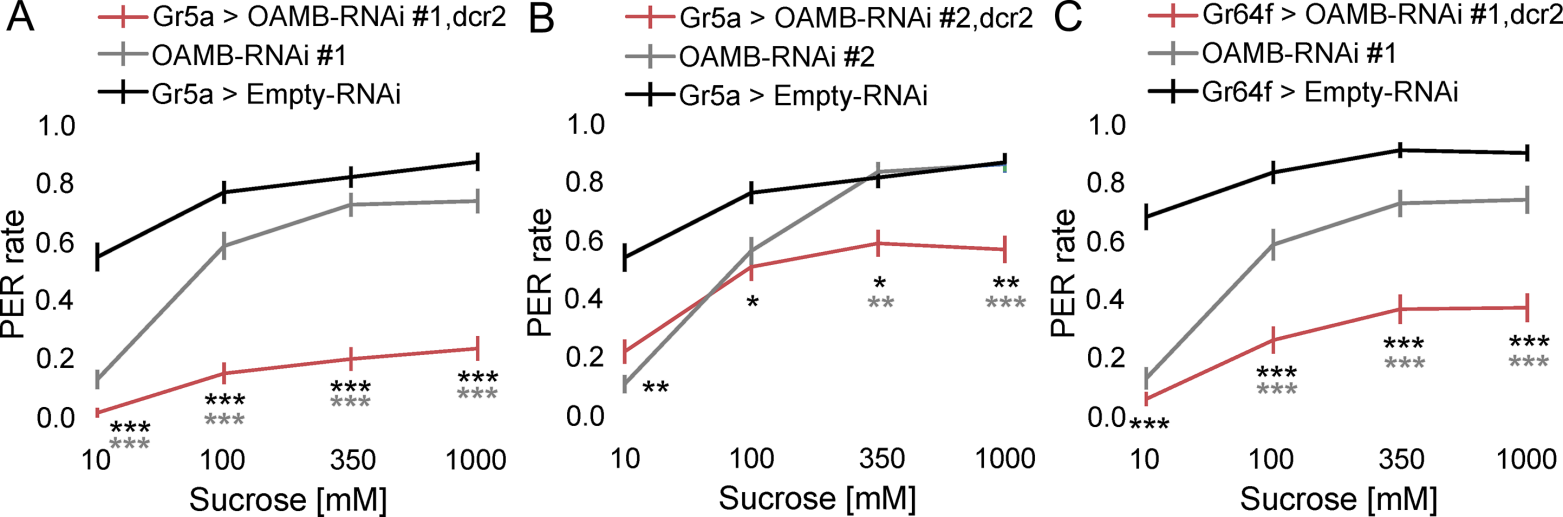
The octopamine receptor OAMB modulates sugar-sensing GRN responses. (A-C) Flies expressing distinct OAMB-RNAi (*UAS-OAMB-RNAi* #1: BDSC #31233; *UAS-OAMB-RNAi* #2: BDSC #2861) in sugar-sensing GRN neurons were tested for PER to 10mM, 100mM, 350mM, 1M sucrose. n = 102 (E, F) and 80 (G) flies, mean ± SEM, Kruskal-Wallis 1-way ANOVA with Bonferroni correction. *p<0.05, **p<0.01, ***p<0.001. Pairwise comparisons to each control, asterisk colors indicate which genetic controls were compared to the experimental group.

We hypothesized that if OA-VPM4 is presynaptic to the sugar-sensing GRN axons and modulates their function via OA, sugar-sensing GRNs should express OA receptors. Therefore, we tested whether one or more of the five OA receptors in *Drosophila* act in sugar-sensing GRNs to modulate proboscis extension, by knocking down receptors with RNAi and testing the effects on proboscis extension to sucrose. PER to different sucrose concentrations was measured in flies expressing each RNAi (two to four independent RNAi lines per receptor) specifically in sugar-sensing GRNs. Of five known OA receptors—Octβ1R, Octβ2R, Octβ3R, OAMB, and Tyr-OctR [29–33], only knocking down OAMB, an alpha-adrenergic-like G-protein-coupled receptor, robustly decreased PER (Fig 5; S4 Fig). Differences in the effectiveness of RNAi knockdowns may contribute to behavioral variation in the RNAi experiments and concentration-dependent inconsistencies. OAMB is likely excitatory as it increases calcium upon activation [29,31]. These results suggest that OAMB promotes the activity of sugar-sensing GRNs.

**Fig 6:**
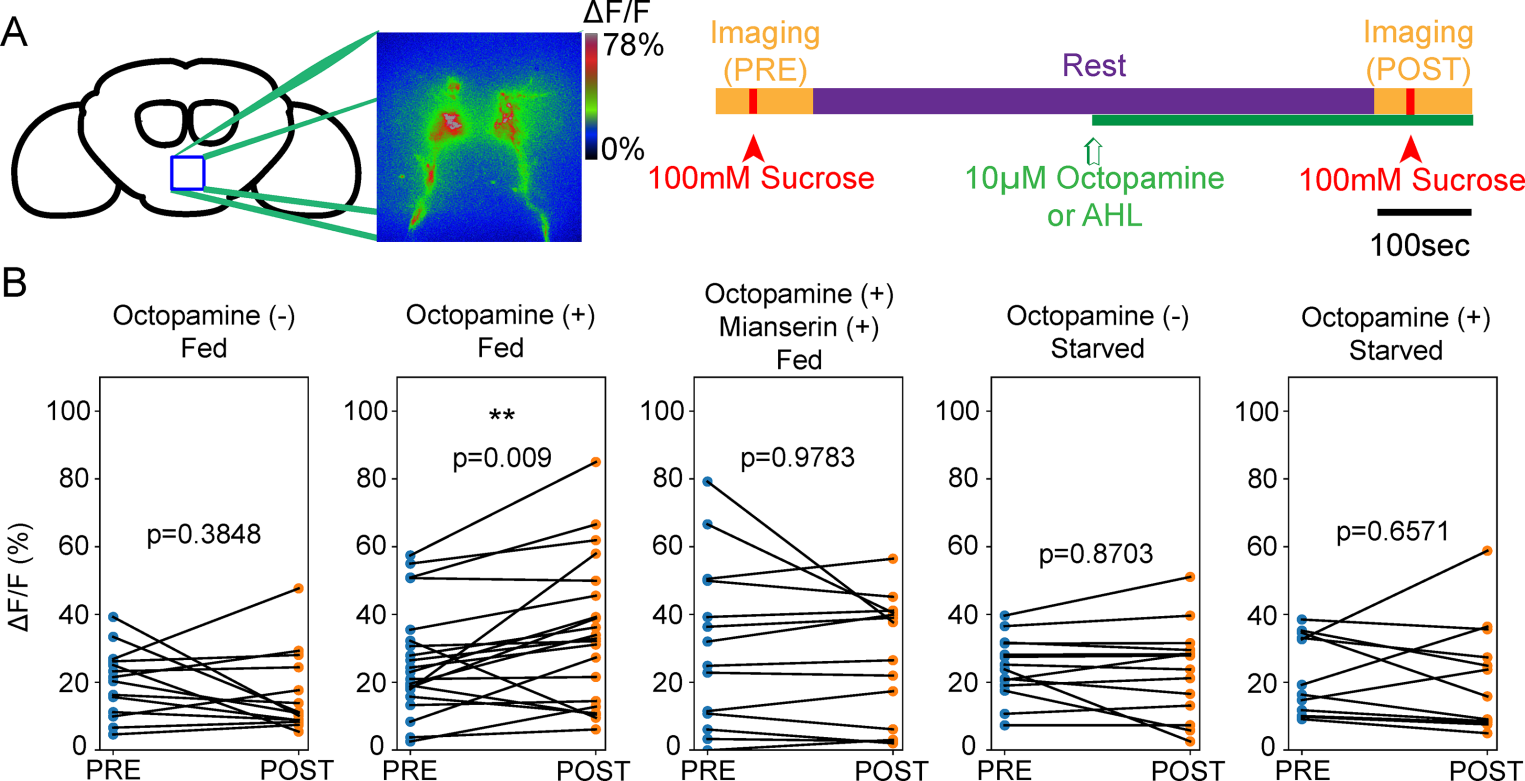
Octopamine potentiates sugar-sensing GRNs responses in satiated flies. (A) Schematic of the calcium imaging experiments monitoring sensory responses to sucrose before and after OA application to the whole brain in a live fly preparation. First, calcium increases in response to 100mM sucrose solution on the proboscis were examined [PRE]. Then, the fly rested for 10 min and 10μM OA was provided to the whole brain at the 5 min time point. Finally, calcium responses to 100mM sucrose on the proboscis [POST] were examined. (B) In fed flies, OA enhanced calcium responses in sugar-sensing GRNs to 100mM sucrose, while control flies with no OA application showed no difference in [PRE] and [POST]. Co-application of 10μM mianserin, an OA receptor antagonist, and OA abolished the potentiation. Applying OA did not induce potentiation in starved flies. Each data point represents the max ΔF/F of a single neuron. n = 7–12 flies each. Wilcoxon signed-rank test for clustered data. **p<0.01.

Does OA directly modulate sugar-sensing GRN activity? If sugar-sensing GRNs respond to OA through OAMB, we predicted that OA application should increase the taste responses of these neurons. To test this hypothesis, we compared the calcium responses of sugar-sensing GRN axons triggered by 100mM sucrose delivered to the proboscis, before and after applying OA to the whole brain (Fig 6). Application of 10μM OA was sufficient to modestly potentiate tastant-driven calcium responses in these neurons in fed flies, but not in starved flies. Blocking the function of OAMB using the receptor antagonist Mianserin [34] abolished the octopamine-dependent calcium enhancement. These results suggest that OA indeed potentiates the taste response in the sugar-sensing GRNs, likely via OAMB.

## Discussion

Feeding behavior must be tightly regulated to coordinate consumption and satisfy current metabolic needs. Here, we show that activating OA-VPM4 promotes proboscis extension to sucrose, whereas decreasing its activity reduces PER. Suppressing OA synthesis in OA-VPM4 also decreases PER. OA-VPM4 has axonal arbors near sugar sensory projections, suggesting that OA-VPM4 may modulate sensory responses. Consistent with this, knocking down a specific class of OA receptors, OAMB, in sugar sensory neurons, reduces proboscis extension. These studies demonstrate that OA synthesis in OA-VPM4 and OA detection by sugar sensory neurons both increase the probability of feeding initiation to promote feeding behavior, providing a deeper understanding of octopaminergic feeding control.

**Fig 7:**
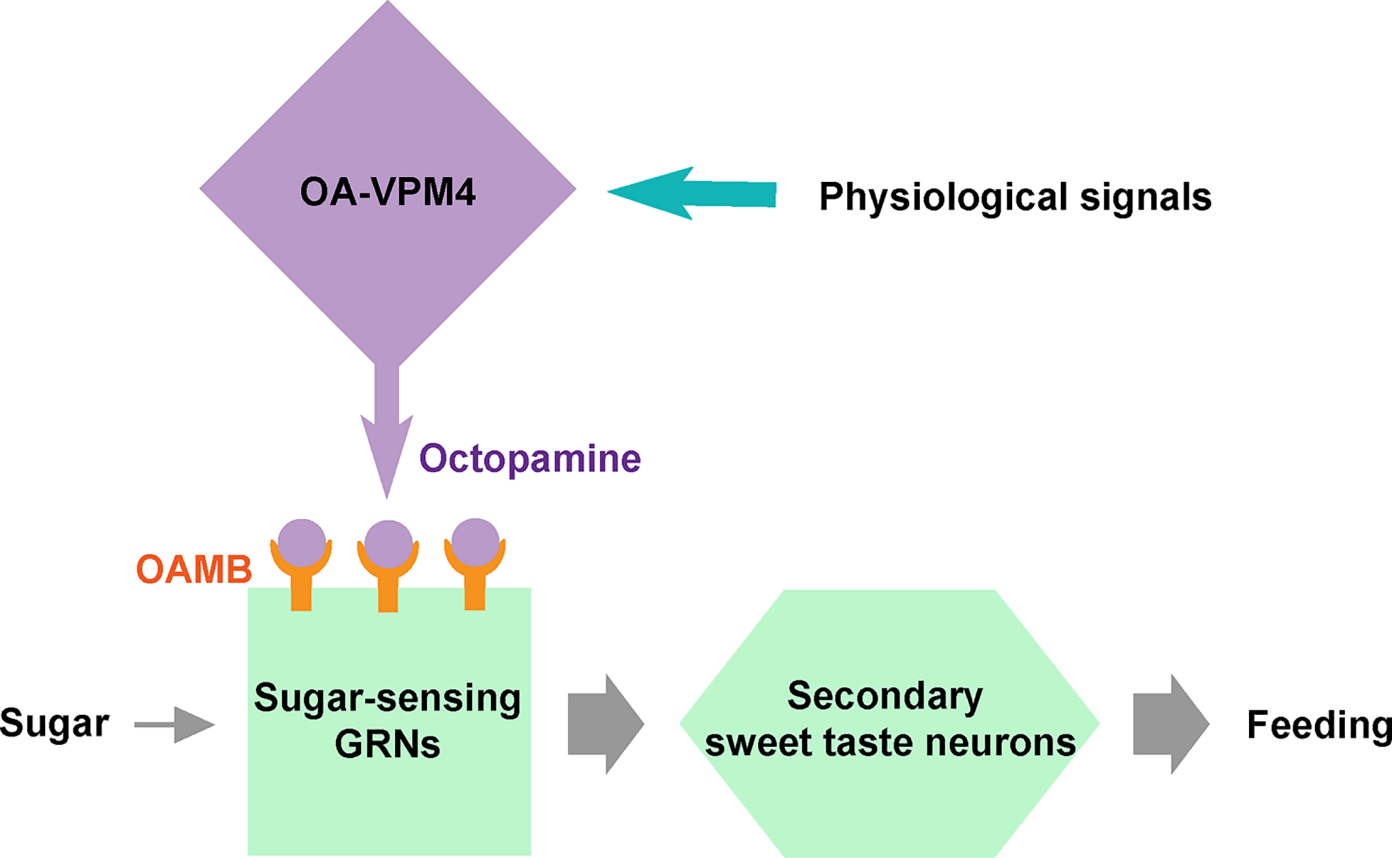
Model: OA-VPM4 enhances feeding by potentiating sugar-sensing GRNs via an octopaminergic pathway. Schematic of the model. OA-VPM4 releases OA in response to physiological signals (i.e., hunger or arousal). OA, in turn, activates OAMB on sugar-sensing GRNs and potentiates their responses.

The observations that OA-VPM4 and sugar sensory projections are in close proximity, and that OA synthesis in OA-VPM4 and OA detection by sugar-sensing GRNs both promote proboscis extension, suggest that OA-VPM4 may directly feed onto sugar sensory axons to regulate their activity (Fig 7). Although this is the most parsimonious model, functional tests of connectivity between OA-VPM4 and axons of the sugar-sensing GRNs will be necessary to substantiate it. There are multiple OA neurons projecting within SEZ [20,35] and it is possible that other OA neurons project onto sugar-sensing GRNs and modulate their activity. In addition, OA-VPM4 arborizes widely throughout the protocerebrum, including the mushroom bodies, arguing that it modulates multiple brain areas and serves additional functions beyond its role influencing PER described here.

We propose that OA release from OA-VPM4 has a specific role in enhancing sensory responses to sugar to promote feeding, as activating OA-VPM4 promotes PER, OA potentiates sugar sensory responses, and loss of OAMB in sensory neurons decreases PER. We find that OA potentiates sensory responses in fed flies but not starved flies, suggesting that OA levels may be saturated in starved animals or that sensory responses may be maximal and not subject to further OA regulation. The physiological conditions under which OA is regulated to promote feeding initiation are unknown. OA has numerous functions in insects as an alerting signal, with roles in motivation, arousal, and behavioral initiation, among others [36]. Thus, while it is possible that OA influences sugar sensory activity in response to nutrient depletion, it is also possible that OA release is an arousal or stress signal. Regardless, our studies argue that the effect of OA on sugar sensory responses is to increase activity, promoting PER probability.

OA-VPM4 neurons promote acceptance of sugars, suggesting that they are more active when flies are hungry or aroused. Previous studies showed that different SEZ OA neurons, OA-VLs, are more active when flies are satiated and modulate bitter GRNs to increase rejection of bitter food [14]. As different subsets of OA neurons influence responses to bitter compounds versus sugars, they may provide local control of OA release to precisely regulate taste sensitivity and feeding behavior. Our studies suggest a scenario in which sweet and bitter taste sensitivity are independently modulated by OA neurons acting locally and specifically on different classes of gustatory axons in the context of different internal states.

Previous studies have shown that dopamine acting on a specific dopamine receptor, DopEcR, expressed in sugar sensory neurons, potentiates responses to sugar in starved *Drosophila* [12,15]. Thus, both dopamine and octopamine increase the sensory response to sugars, demonstrating multiple, independent tuning systems. The responsiveness of sensory neurons to sucrose is differently modulated based on nutritional state [12,15], with the potential for differential regulation based on factors such as the specific nutrients depleted and the accumulated deprivation time. In addition, arousal, motivation, or circadian time might modulate sensory responses based on increased metabolic need. The finding that both dopamine and octopamine influence sensory responses argues that this first step of sensory processing is a critical node for regulation, consistent with several studies of chemosensory processing in flies and *C*. *elegans* [37–42]. The observation that multiple modulators influence sensory responses argues the importance of filtering incoming sensory information prior to propagation through brain networks.

## Materials and methods

### Fly stocks

Flies were grown on standard molasses fly food [43]. For optogenetic experiments, retinal was included in the food (0.4 mM all-trans retinal, Sigma). Flies were raised at 25°C, except for experiments involving temperature-sensitive dTrpA1, in which flies were raised at 19°C. The transgenic flies used were *891-Gal4* (Clandinin collection [44]); *MB021B-Gal4* [22]; *Tdc2-LexA* [45]; *Gr64f-Gal4* [46]; *Gr5a-Gal4* [47]; *Gr64f-LexA* [48]; *UAS-CD8*::*GFP* [49]; *UAS-CD8*::*tdTomato* [50]; *UAS-HA*, *LexAop-V5* (BSC#64092); *LexAop-CD2*::*GFP* [51]; UAS-CD4::GFP [5]; *UAS-DenMark* [28]; *UAS-synaptogmin-eGFP* [27]; *UAS-GCaMP6s* [52]; *UAS-dTrpA1* [23]; *UAS-GtACR1* [53]; *UAS-Dcr2* (BSC# 24644); *UAS-Octβ1R-RNAi* (BSC#50701; BSC#58179); *UAS-Octβ2R-RNAi* (BSC#50580; BSC#34706); *UAS-Octβ3R-RNAi* (BSC#31108; BSC#62283); *UAS-OAMB-RNAi* (BSC#2861; BSC#31233); *UAS-Tyr-OctR-RNAi* (BSC#26876); *UAS-Tdc2-RNAi* (BSC# 25871); *UAS-Tβh-RNAi* (BSC# 27667); *UAS-pdfR-RNAi* (BSC#38347); *UAS-Empty-RNAi* (BSC#36303); *UAS-nSyb-RNAi* (BSC#31983); *MCFO-5* (BSC#64089). BSC# = Bloomington Stock Center number.

### Immunohistochemistry

Immunohistochemical staining was performed as previously described [54]. The primary antibodies used were rabbit anti-GFP (Invitrogen, Carlsbad, CA 1:1000), mouse anti-GFP (Invitrogen 1:1000), mouse anti-nc82 (Developmental Studies Hybridoma Bank, Iowa City, IA 1:500), rabbit anti-RFP (Clonetech, Mountain View, CA 1:500); rabbit α-HA Tag (Cell Signaling Technologies 1:300), DL550 mouse α-V5 (Bio-Rad 1:500), chicken anti-GFP (Life Technologies, 1:500). The secondary antibodies were (all Invitrogen at 1:100): 488 anti-rabbit, 488 anti-mouse, 488 anti-chicken, 568 anti-rabbit, 568 anti-mouse. All images were acquired on a confocal microscope. Afterward, brightness and contrast were adjusted with FIJI (ImageJ) software.

### Multi color flip out (MCFO)

*MCFO-5* flies (BSC#64089) were crossed with *MB021B-Gal4*, and the 7–10 days old progeny were antibody stained and imaged following the fly light protocol (multi-color flip out (MCFO) IHC for adult *Drosophila* CNS).

### Calcium imaging experiments

#### Monitoring OA-VPM4 response to sucrose

Flies 4–7 days old expressing two copies of *UAS-GCaMP6s* and one copy of *UAS-CD8*::*tdTomato* under the control of *891-Gal4* were starved for 24 hours and prepared for calcium imaging as previously described [51]. Twenty-two z-planes (1.2 μm thickness each, 100 ms exposure) were scanned for each time point, 40 time points total. At time points 10 and 20, 1M sucrose was delivered to the proboscis; at time point 30, 1M KCl was applied to the brain.

For analysis, we subsetted maximum projection of Z volume. The change in calcium fluorescence (F) was calculated as ΔF/F = F(t)–F(0) / F(0), where F(0) is an average F (5 time points) before each stimulation and F(t) is an average F (5 time points) after the onset of stimulus. Manual ROI selection and fluorescence measures were performed in FIJI (ImageJ) and further analysis was done with R and Graphpad Prism. Representative ΔF/F heat map images were generated in FIJI (ImageJ).

#### Octopamine application experiments

Flies expressing three copies of *UAS-GCaMP6s* and one copy of *UAS-CD8*::*tdTomato* under the control of *Gr64f-Gal4* were generated to examine calcium transients and define the imaging volume. 7–10 days old flies were prepared for calcium imaging as previously described [55], with the brain dissected and immersed in modified artificial *Drosophila* hemolymph (AHL) (10mM sucrose and 5mM trehalose substituted with 15mM ribose). Sugar-sensing GRN axons (*Gr64f-Gal4* expressing) were imaged on a fixed-stage 3i spinning disk confocal microscope with a piezo drive and a 20x water objective (1.6x optical zoom), as previously described [55]. The location of sugar-sensing GRN axons was determined with a 561nm laser before imaging calcium transients with a 488nm laser. Each z-plane with 1.0μm thickness was scanned in 100msec and 14 z-planes (15μm) were scanned in total for each time point. Calcium transients in sugar-sensing GRNs were imaged (Fig 6B—PRE) during proboscis stimulation with sucrose (100mM; 7~8sec stimulation) [56]. After imaging, flies rested in dark for 10min. At 5min, 1μl of 2mM octopamine was applied, 10μM final concentration. After Z movement correction, calcium transients of sugar-sensing GRNs upon 100mM sucrose stimulation were imaged as above (Fig 6B—POST).

A maximum projection of Z volume was used for analysis. The calcium fluorescence (F) change was calculated as ΔF/F = F(t)–F(0) / F(0). F(0) is an average F (five time points, ~13sec) before taste stimulation. Max F is the maximum calcium response (max ΔF/F) within five time points after initial stimulation, which generally occurs during the stimulation period. Manual ROI selection, X-Y movement correction, and fluorescence measures were performed in FIJI (ImageJ) and further analysis was executed with Python and R.

### Proboscis extension response (PER)

PER was performed as previously described [57], except that each animal was considered a data point and was categorized as responding 0, 1, 2, or 3 times. 7–10 days old flies were anesthetized with CO_2_, glued dorsal side down on a glass slide, and allowed to recover in a humid chamber for 2 hours before behavioral tests. Flies were fed with water to satiation before testing, then given a series of sucrose concentrations (10mM, 100mM, 350mM, 1M) on their tarsi at ~3 min intervals. These concentrations were selected because they induce different probabilities of proboscis extension [12,58]. Tarsi were washed and flies were allowed to drink water between each concentration test. For thermogenetic experiments, flies were starved at 19°C in empty vials containing wet kimwipes for 24 hours. They were placed on a heat pad (31°C) ~5min before and during testing, except for room temperature controls (21°C). For optogenetic experiments, flies were raised in vials wrapped in tin foil until testing. Flies were moved into retinal-reinforced food (0.4mM all-trans retinal) 48 hours prior to the experiment, and starved on wet kimwipe with 0.4 mM all-trans retinal 24 hours before the experiment. Flies were mounted on glass slides as described above and photo-stimulated on the customized LED light pad with 530nm wavelength while PER was examined.

PER rate was calculated as:

PERrate=∑PERscoreofeachfly/(Numberofflies*3)

PER score of each fly is an integer value varying from 0 to 3, indicating how many times flies extended the proboscis to 3 stimulations.

### Statistics

For PER assays comparing three experiment groups, Kruskal-Wallis 1-way ANOVA test (non parametric) with Bonferroni correction for multiple comparisons was used (Fig 2D and 2H; Fig 3B and 3C; Fig 5E, 5F and 5G). Mann-Whitney-U test (non-parametric) was used for assays comparing two experimental groups (Fig 2A–2C and Fig 2E–2G; S4 Fig). For calcium imaging with octopamine application (Fig 6B), Wilcoxon signed-rank test modified for clustered data was used to compare pre and post octopamine application [59–61].

## Supporting information

S1 Fig OA-VPM4 neurons are octopaminergic. (A) Double labeling of *891-Gal4* neurons (green, HA) and *Tdc2-LexA* neurons (magenta, V5). Scale = 50μm.
(TIF)

S2 Fig OA-VPM4 does not respond to proboscis stimulation with sucrose. (A) Schematic of brain area monitored (left) and ΔF/F images for an example brain showing calcium-induced GCaMP6s fluorescent changes in response to 1M sucrose delivered to the fly proboscis of a live fly (middle) or 1M KCl applied to the bath (right).
(B) GCaMP6s change (ΔF/F) upon 1M sucrose stimulation (two presentations) followed by 1M KCl stimulation for 6 brains, mean (dark line), SEM (grey shade).
(C) Maximum GCaMP6s change (ΔF/F) for the 6 brains shown in (B), mean ± SEM.
(TIF)

S3 Fig Proximity of OA-VPM4 dendrites and sugar-sensing GRN axons. Double labeling of *UAS-DenMark* expressed by *891-Gal4* (magenta) and axonal projection of sugar-sensing GRNs (green) in SEZ, anterior (A), middle (B), and posterior (C). There is little overlap, suggesting that OA-VPM4 neurons are not postsynaptic to sugar-sensing GRNs.
(TIFF)

S4 Fig Testing the function of octopamine receptors in sugar-sensing GRNs. Flies containing RNAi against different OA receptors expressed in sugar-sensing taste neurons were tested for proboscis extension to sugar (10, 100, 350, 1000 mM). black lines = *UAS-RNAi*; red lines = *Gr5a-Gal4*, *UAS-RNAi*. n = 30–55 flies, mean ± SEM, Mann-Whitney-U test, *p<0.05, **p<0.01, ***p<0.001.
(TIF)
