# Supplementary figures and images for: A subset of octopaminergic neurons that promotes feeding initiation in Drosophila melanogaster
Source: PLoS One. 2018 Jun 27;13(6):e0198362. doi: 10.1371/journal.pone.0198362 (PMC6021039; doi:10.1371/journal.pone.0198362)

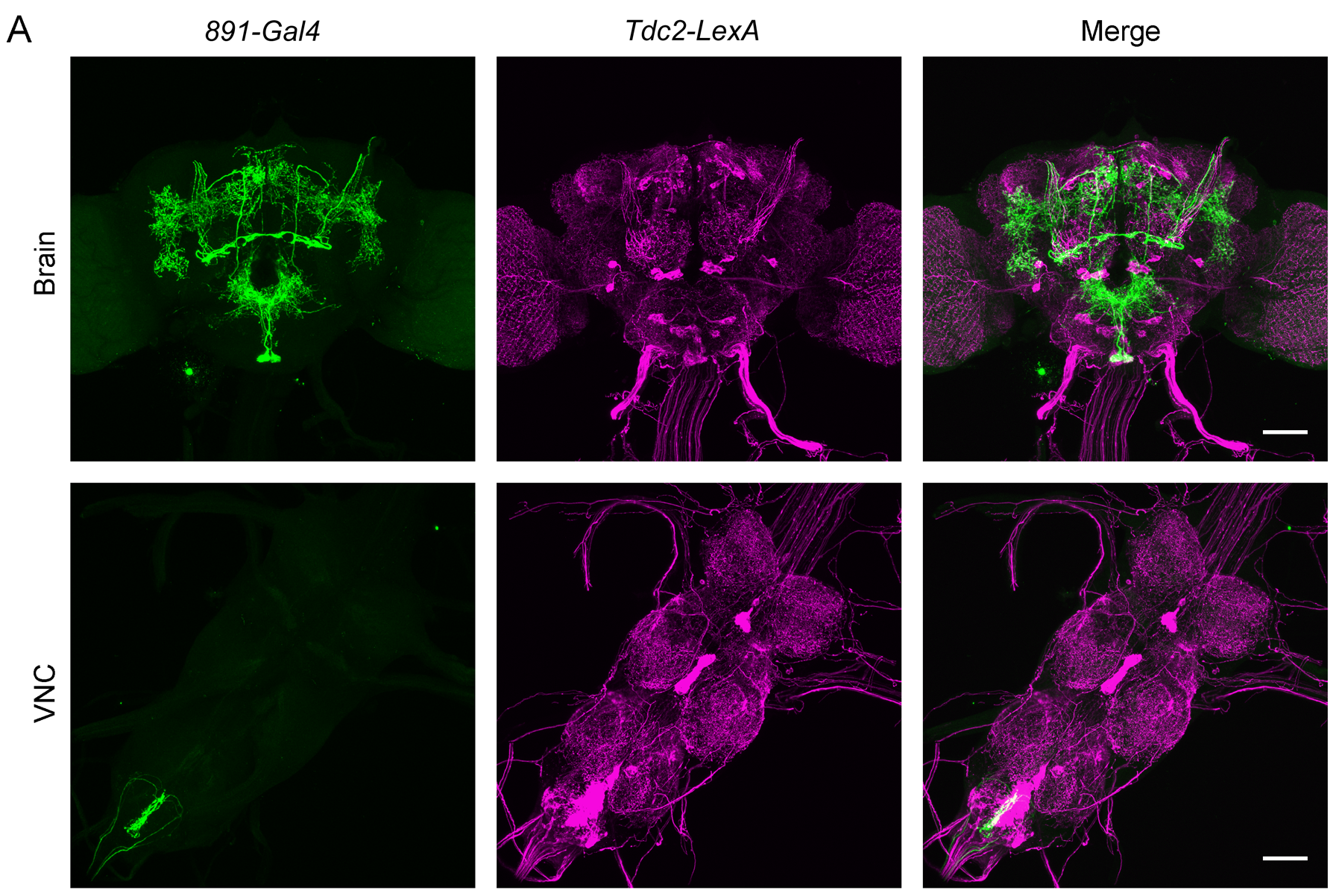

Supplement: S1 Fig — (A) Double labeling of 891-Gal4 neurons (green, HA) and Tdc2-LexA neurons (magenta, V5). Scale = 50μm. (TIF) [file pone.0198362.s001.tif]

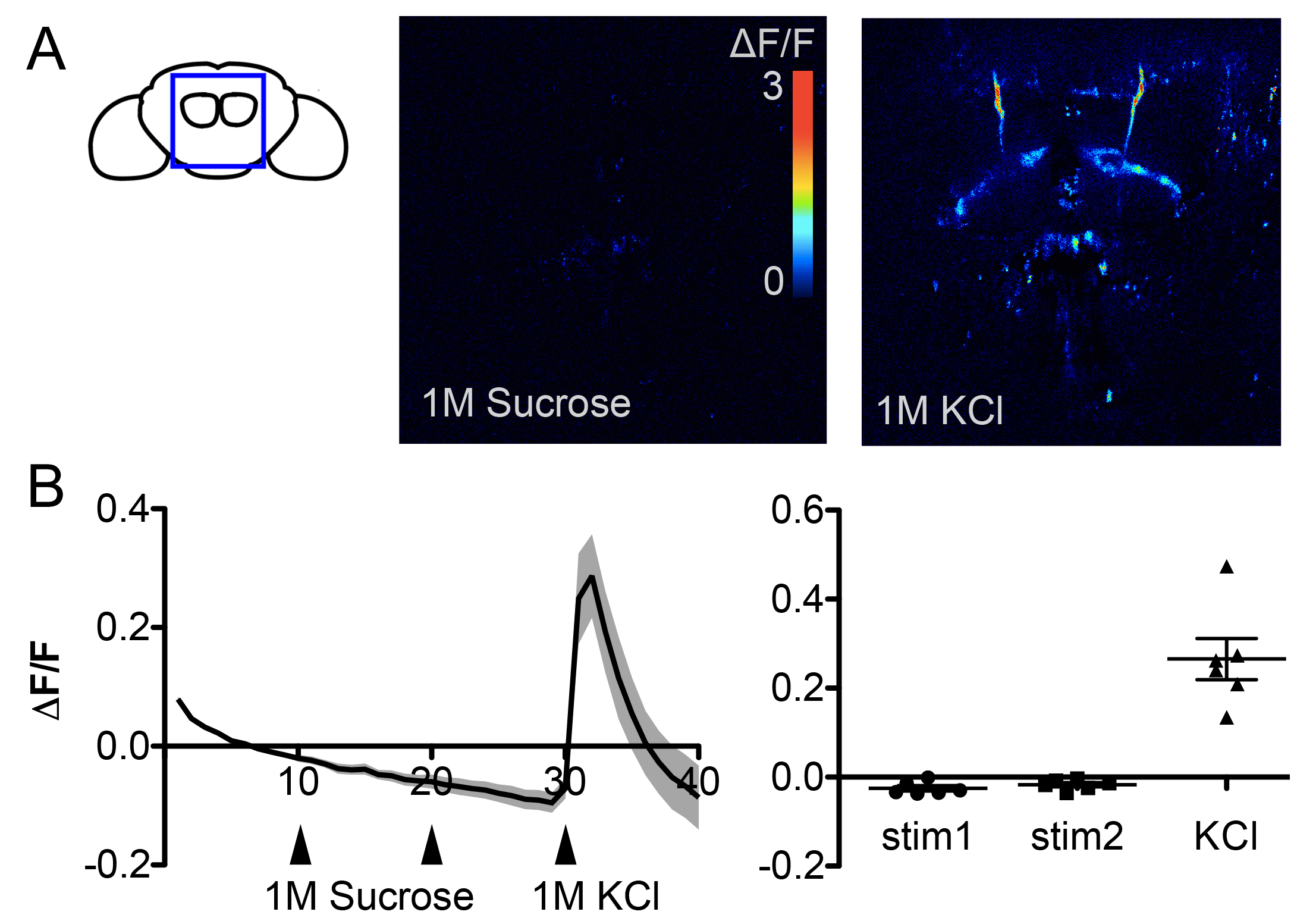

Supplement: S2 Fig — (A) Schematic of brain area monitored (left) and ΔF/F images for an example brain showing calcium-induced GCaMP6s fluorescent changes in response to 1M sucrose delivered to the fly proboscis of a live fly (middle) or 1M KCl applied to the bath (right). (B) GCaMP6s change (ΔF/F) upon 1M sucrose stimulation (two presentations) followed by 1M KCl stimulation for 6 brains, mean (dark line), SEM (grey shade). (C) Maximum GCaMP6s change (ΔF/F) for the 6 brains shown in (B), mean ± SEM. (TIF) [file pone.0198362.s002.tif]

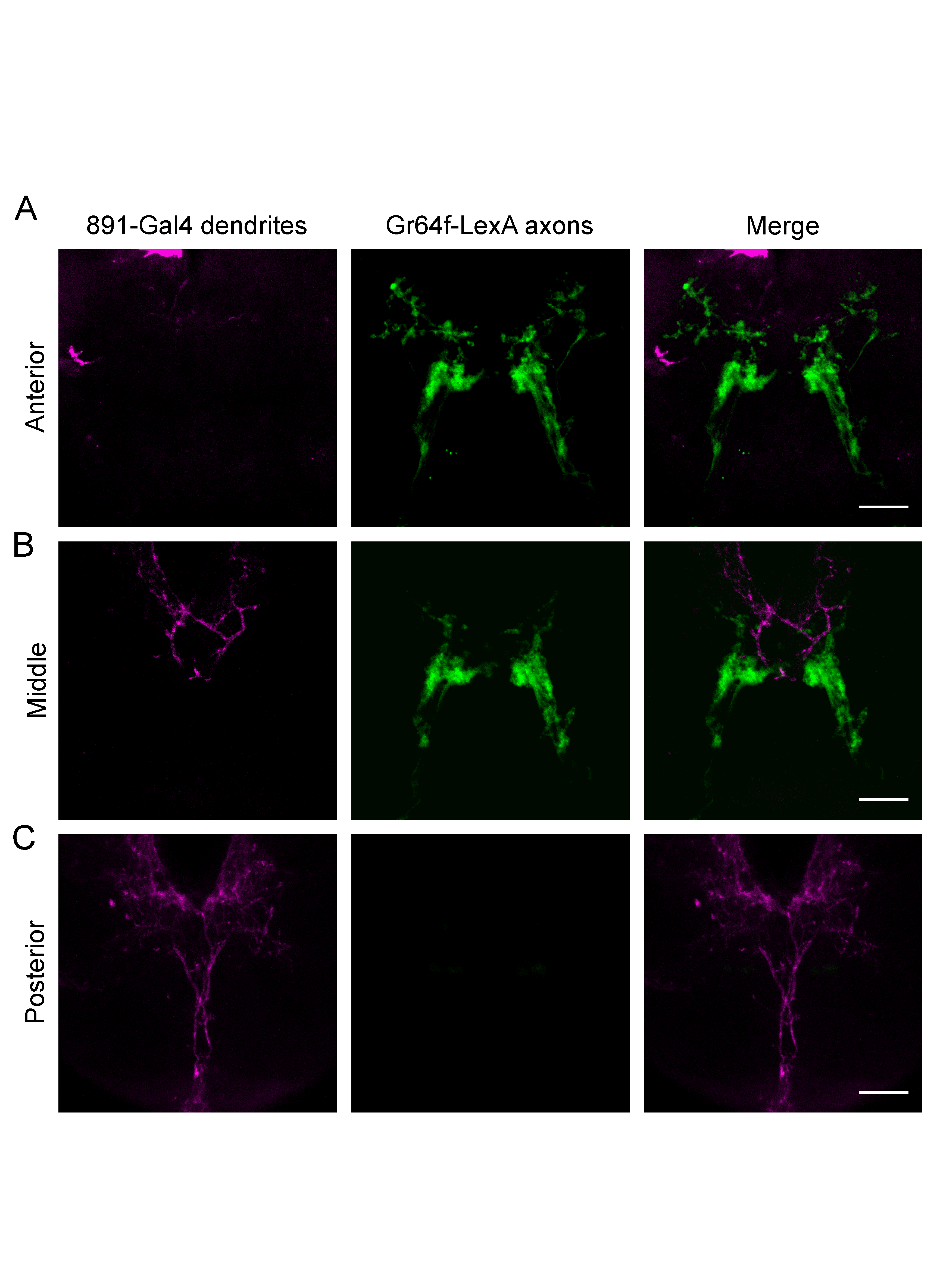

Supplement: S3 Fig — Double labeling of UAS-DenMark expressed by 891-Gal4 (magenta) and axonal projection of sugar-sensing GRNs (green) in SEZ, anterior (A), middle (B), and posterior (C). There is little overlap, suggesting that OA-VPM4 neurons are not postsynaptic to sugar-sensing GRNs. (TIFF) [file pone.0198362.s003.tiff]

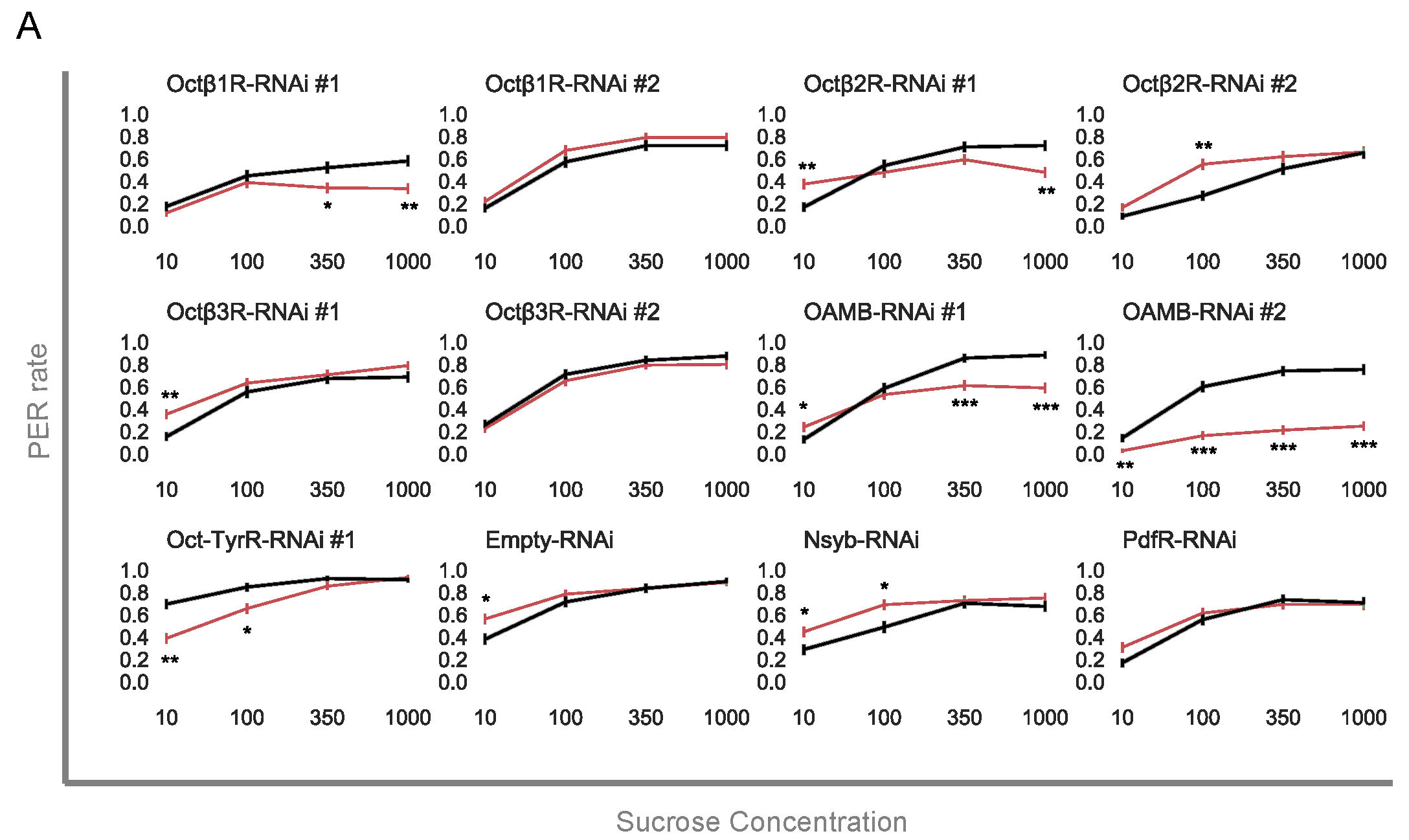

Supplement: S4 Fig — Flies containing RNAi against different OA receptors expressed in sugar-sensing taste neurons were tested for proboscis extension to sugar (10, 100, 350, 1000 mM). black lines = UAS-RNAi; red lines = Gr5a-Gal4, UAS-RNAi. n = 30–55 flies, mean ± SEM, Mann-Whitney-U test, *p<0.05, **p<0.01, ***p<0.001. (TIF) [file pone.0198362.s004.tif]
